# Relation between left atrial structure and lacunar infarction in patients with hypertension

**DOI:** 10.18632/aging.103697

**Published:** 2020-09-10

**Authors:** Ting Sun, Tong Xie, Alian Zhang, Li Fan, Zuojun Xu, Xin Chen, Zhicheng Fan, Changqian Wang

**Affiliations:** 1 Department of Cardiology, Shanghai Ninth People’s Hospital, Shanghai JiaoTong University School of Medicine, Shanghai 200025, China; 2 Department of Intensive Care Unit, Shanghai Xuhui District Central Hospital, Shanghai 200031, China

**Keywords:** hypertension, lacunar infarct, left atrium volume, ratio of left atrial diameter to left ventricular diameter

## Abstract

A lacunar infarction (LACI) can cause damage to the surrounding brain tissue and place an individual at greater risk for future major stroke. LACI is associated with hypertension and hypertension is associated with left atrial enlargement. It is important to identify a high-risk patient who is more vulnerable to suffering a LACI in hypertensive group. So, we studied whether left atrium size is an independent risk predictor for LACI in hypertensive patients. We performed cross-sectional analysis of 365 patients with hypertension at Shanghai Ninth People’s Hospital from January 2016 to January 2017. The results showed that left atrial diameter(LAD), left atrial volume (LAV) and the ratio of left atrial diameter to left ventricular diameter (LAD/LVD) were significantly associated with LACI in hypertensive patients. Based on the ROC curve analysis, the area under the ROC curve (AUC) of LAV used to predict LACI was 0.737 (95% CI: 0.686 – 0.788), and the AUC of LAD/LVD was 0.784 (95% CI: 0.737 – 0.830). The optimal cut-off value for LAV was 30.14, and the sensitivity and specificity were 72% and 63%, respectively. The optimal cut-off value for LAD/LVD was 0.757, and the sensitivity and specificity were 77% and 70%, respectively. LAV or LAD/LVD played an important role in LACI with hypertension and could be an independent risk factor in hypertensive patients.

## INTRODUCTION

Lacunar infarct (LACI), a small deep infarct that results from occlusion of a penetrating artery, accounts for approximately one-quarter of all ischemic strokes [1]. The corresponding lesion in the brain issue is removed to ultimately form a small cavity with a diameter of no more than 20 mm, although the majority of which are 2 to 4 mm. The clinical manifestations of LACI are not typical. Most of them have occult diseases/onsets, some can show acute or subacute onsets, and many cause no disturbance of consciousness. A silent LACI is one type of silent stroke that usually shows no identifiable outward symptoms, and is thus termed "silent" [2]. Even though the pathophysiology is presumably the same as LACI, a silent LACI is considered a stroke, and individuals are often completely unaware they have suffered a stroke due to the lack of classic stroke symptoms. However, silent LACI can also cause damage to the surrounding brain tissue lesions and affect various aspects of a person’s mood, personality, and cognitive functioning. The lesion can often be visibly detected via neuroimaging techniques such as MRI and computerized axial tomography (CT scan). Any type of LACI can place an individual at greater risk for future major stroke and increase the risk of death, which is mainly related to cardiovascular events [3]. Hence, prevention is likely to play a large part in therapeutic interventions targeting this stroke subtype.

With the rising awareness of the multiple risk factors for LACI, early detection of those risk factors, especially those related to cardiovascular diseases, for the prevention of target organ damage has become a reality. The risk factors for LACI are advanced age, chronic hypertension, smoking and diabetes mellitus [4]. Among these known risk factors, hypertension, the leading cause of LACI, affects 50% of Chinese adults aged 35-75 years [5]. Hypertension was the main cerebrovascular risk factor only for lacunar infarct and atherothrombotic infarction, that is, ischemic stroke associated with small- and large-artery disease [6]. Arteriolar hyaline degeneration, fine atherosclerosis and fibrinoid necrosis caused by hypertension are important pathogeneses of LACI. Left atrial enlargement is one of the early signs of heart disease caused by hypertension, which is mainly due to decreased filling function and compliance in the left ventricle [4]. The long-term overload of left atrium will inevitably lead to compensatory hypertrophy of the atrial muscle, which eventually causes left atrial enlargement. Changes in atrial function can induce increased atrial pressure load and stasis of blood flow, thus causing intimal injury and turbulence in the heart chamber and leading to thrombosis [7]. This is the pathological basis for the occurrence of stroke. Mazouz et al. found that a left atrial diameter (LAD) >40mm can independently predict the formation of a left atrial thrombus [8]. Data from the Framingham Heart study showed that for every 10 mm increase in left atrial size, the relative risk of stroke was 2.4 in males and 1.4 in females after multivariate adjustment [9]. Based on a retrospective cohort study, Barnes et al. found that for individuals without atrial fibrillation, a left atrial volume (LAV) of 32 ml/m2 or more is an independent predictor of the first occurrence of ischemic stroke [10]. They believe that LAV can be a surrogate indicator of overall risk, including cardiovascular risk and death risk. As seen above, left atrium size is inextricably linked with stroke.

The prevalence rate of hypertension in China is relatively high; however, patients’ awareness about hypertension treatment and control is poor. From 1991 to 2011, approximately one-fifth of Chinese adults were hypertensive, but only 17.5% of hypertension was controlled. Peng J et al studied 480 elderly hypertensive Chinese patients and found that left atrial enlargement is independently associated with a longer duration of hypertension in elderly hypertensive patients with preserved LVEF [5]. Since there is a positive association between left atrial enlargement and hypertension and hypertension is an important pathogenesis of LACI, we hypothesize that LACI is associated with left atrial size in hypertensive patients. Therefore, we used noninvasive echocardiography to measure left atrial size and left ventricular dimension in hypertensive patients and to assess the relationship between left atrial size and the incidence of LACI. By assessing the left atrium size with echocardiography, we can predict high-risk populations that are prone to LACI and provide them early intervention.

## RESULTS

### Clinical characteristics

This study included 365 participants (170 males and 195 females; mean age 75.3 ± 10.5 years, minimum 45, maximum 96). Of the 365 participants, 180 participants had LACI. Among 180 patients with lacunar infarctions, 72 cases were pure motor hemiparesis, 35 cases were pure sensory stroke, 31 cases were sensorimotor stroke, 11 cases were ataxic-hemiparesis, 7 cases were dysarthria-clumsy hand, 5 cases were atypical lacunar syndromes and 19 cases were no identifiable outward symptoms. Brain MRI or CT scanning studies showed that the lesions of LACI were mainly located in the basal ganglia area (131/180, 72.7%), with a small proportion in the thalamus (33/180, 18.3%), corona radiata (9/180, 5.0%) and cerebral cortex (7/180, 3.9%). In the non-LACI group, there were transient ischemic attacks (TIAs, n= 22), hemorrhagic stroke (n= 5), coronary heart disease (CHD, n= 117), diabetes (n= 59), and diabetes (n= 18).

The clinical characteristics of the study patients are presented in Table 1. There were no significant differences in sex, hypertension classification, coronary heart disease, or diabetes mellitus between the LACI and non-LACI groups. In all patients, 12-lead electrocardiogram was performed within the first day of hospital admission. The rate of echocardiographic studies was not difference between the two groups (Table 1). Premature beats were observed in 35.6% of patients on electrocardiogram, and then a Holter examination was recorded and showed no serious arrhythmia in these patients. Compared withthose in the non-LACI group, the patients in the LACI groupwere older and had a longerduration of hypertension (*P*< 0.01), which indicates that with increasing age and hypertension duration, the incidence of cavity infarction was higher.

**Table 1:** Clinical characteristics and cardiac ultrasound indexes of studied sample.

**Variable**	**Non-LACI (n=185)**	**LACI (n=180)**	***t/ U/χ^2^* Value**	***P* Value**
Age, y	73.11 ± 10.40	77.52 ± 10.23	-4.080	0.000
Gender (M,%)	46.5 (86/185)	46.7 (84/180)	0.001	0.972
HTN Classification, %			0.159	0.690
Grade I	45.4 (84/185)	43.3 (78/180)		
Grade II and III	54.6 (101/185)	56.7 (102/180)		
HTN time,%			11.258	0.004
<10 years	34.6 (64/185)	21.7 (39/180)		
10-20 years	35.7 (66/185)	33.3 (60/180)		
>20 yeaes	29.7 (55/185)	45.0 (81/180)		
CHD, %	63.2 (117/185)	66.1 (119/180)	0.328	0.567
Diabetes, %	31.9 (59/185)	33.3 (60/180)	0.086	0.769
HR,bpm	76.35 ± 8.28	74.12 ± 9.99	-1.008	0.313
LAV,ml	29.66 ± 3.98	35.45 ± 8.79	-7.831	0.000
LAD,mm	37.26 ± 4.72	42.16 ± 7.30	-7.091	0.000
IVS,mm	10.68 ± 1.61	11.25 ± 2.21	-2.804	0.005
LVD,mm	51.52 ± 6.79	50.10 ± 7.80	1.856	0.064
LVPW,mm	10.60 ± 1.49	10.97 ± 1.76	-2.149	0.032
LVEF,%	56.69 ± 7.85	55.25 ± 7.80	1.760	0.079
LAD/LVD	0.73 ± 0.09	0.85 ± 0.14	-9.373	0.000

LACI, Lacunar Infarction; HTN, Hypertension; M, Male; CHD, Coronary heart disease; HR, Heart Rate; LAV, left atrial volume; LAD, left atrial dimension; IVS, interventricular septal thickness at end-diastole; LVD, left ventricular dimension; LVPW, left ventricular posterior wall thickness at end-diastole; LVEF, left ventricular ejection fraction.

### Comparison of cardiac ultrasound indexes of patients in LACI and non-LACI groups

Table 1 shows the cardiac ultrasound indexes of patients in the LACI and non-LACI groups. The patients in the LACI group had a larger left atrial dimension (LAD), a greater left atrial volume (LAV), a thicker interventricular septal thickness (IVS) and left ventricular posterior wall thickness (LVPW) than those in the non-LI group. The ratio of left atrial diameter to left ventricular diameter (LAD/LVD) was higher in the LACI group than in the non-LACI group. However, the left ventricular dimension (LVD) and left ventricular ejection fraction (LVEF) were not different between the two groups.

### Comparison of cardiac ultrasound indexes and clinical characteristics of patients with different grades and courses of hypertension

Table 2 shows cardiac ultrasound indexes and clinical characteristics of patients in the grade I hypertension group (n= 162) and grade II hypertension and above group (n= 203). None of the indexes were different between the two groups (*P*> 0.05). Table 3 shows cardiac ultrasound indexes and clinical characteristics of patients with different courses of hypertension. There were no significant differences in sex, coronary heart disease, or diabetes mellitus among these groups. Age was obviously increased in patients with a history of hypertension longer than 10 years compared to those with a history of hypertension under 10 years. There was larger LAD, LAV LAD/LVD and thicker IVS and LVPW measurements in hypertension patients with over 2 years of hypertension history than in patients who had a shorter hypertension history, but LVD and LVEF were not different among the different course groups.

**Table 2:** Cardiac ultrasound indexes and clinical characteristics of patients indifferent grade of hypertension.

**Variable**	**Grade I hypertension (n=162)**	**Grade II hypertension and above (n=203)**	***t/U* Value**	***P* Value**
Age, y	76.33± 9.05	74.44 ± 11.54	-0.901	0.386
Gender (M,%)	47.5 (77/162)	45.8 (93/203)	0.107	0.753
CHD, %	65.4 (106/162)	64.0 (130/203)	0.076	0.826
Diabetes, %	32.7 (53/162)	32.5 (66/203)	0.002	0.967
LAV, ml	31.96 ± 7.59	32.96 ± 7.20	-1.102	0.271
LAD, mm	39.55 ± 6.68	39.78 ± 6.54	-0.323	0.747
IVS, mm	10.83 ± 1.97	11.06 ± 1.93	-1.109	0.268
LVD, mm	51.53 ± 7.13	50.25 ± 7.45	1.657	0.098
LVPW, mm	10.67 ± 1.58	10.87 ± 1.68	-1.155	0.249
LVEF, %	56.23 ± 8.56	55.78 ± 7.25	-0.685	0.494
LAD/LVD	0.77 ± 0.12	0.80 ± 0.13	-1.914	0.056

M, Male; CHD, Coronary heart disease; LAV, left atrial volume; LAD, left atrial dimension; IVS, interventricular septal thickness at end-diastole; LVD, left ventricular dimension; LVPW, left ventricular posterior wall thickness at end-diastole; LVEF, left ventricular ejection fraction.

**Table 3:** Cardiac ultrasound indexes and clinical characteristics of patients in different course of hypertension.

**Variable**	**<10 years (n=103)**	**10-20 years (n=126)**	**>20 years (n=136)**	***F* Value**	***P* Value**
Age, y	69.74 ± 11.58	76.62 ± 7.80	75.28 ± 10.38	23.118	0.000
Gender (M,%)	49.5 (51/103)	47.6 (60/126)	43.4 (59/136)	0.970	0.616
CHD, %	65.0 (67/103)	65.9 (83/126)	63.2 (86/136)	0.209	0.901
Diabetes, %	29.1 (30/103)	34.9 (44/126)	33.1 (45/136)	0.889	0.641
IVS, mm	10.60 ± 2.24	10.85 ± 1.85	11.34 ± 1.73	4.633	0.010
LVD, mm	50.44 ± 7.72	51.03 ± 7.54	50.91 ± 6.86	0.198	0.820
LVPW, mm	10.44 ± 1.66	10.66 ± 1.64	11.16 ± 1.54	6.543	0.002
LVEF, %	56.61 ± 7.69	56.08 ± 8.21	55.40 ± 7.63	0.713	0.491
LAD/LVD	0.76 ± 0.13	0.79 ± 0.14	0.80 ± 0.12	3.259	0.040

M, Male; CHD, Coronary heart disease; LAV, left atrial volume; LAD, left atrial dimension; IVS, interventricular septal thickness at end-diastole; LVD, left ventricular dimension; LVPW, left ventricular posterior wall thickness at end-diastole; LVEF, left ventricular ejection fraction.

### ROC curve analysis for LAV and LAD/LVD

**Figure 1:**
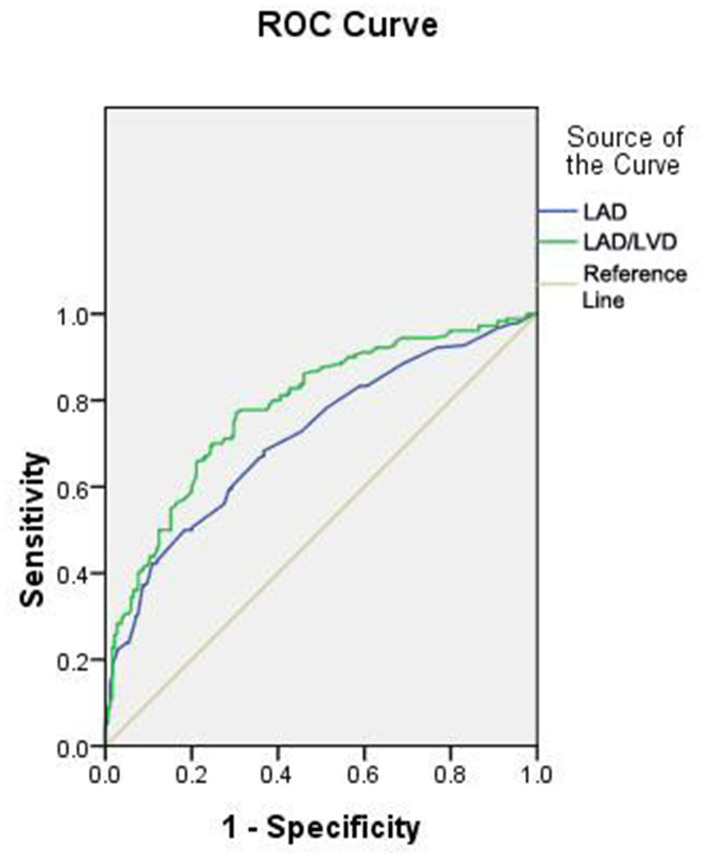
**ROC Curve Analysis of LAV and LAD/LVD.**

To determine the sensitivity and specificity of LAV or LAD/LVD for predicting the occurrence of LACI in hypertension patients, we performed ROC analysis (Figure 1 and Tables 4, 5). The area under the ROC curve (AUC) of LAV used to predict LACI was 0.737 (95% CI: 0.686 – 0.788, *P* = 0.000), and the AUC of LAD/LVD was 0.784 (95% CI: 0.737 – 0.830, *P* < 0.000). Based on the ROC curve analysis, the optimal cut-off value for LAV was 30.14, and the sensitivity and specificity were 72% and 63%, respectively. The optimal cut-off value for LAD/LVD was 0.757, and the sensitivity and specificity were 77% and 70%, respectively.

**Table 4:** ROC curve for various cut-off levels of LAV.

**LAV,ml**	**Sensitivity (95% CI)**	**Specificity (95% CI)**	**DOC**
29.85	0.772 (0.746-0.800)	0.541 (0.515-0.567)	0.513
30.14	0.722 (0.696-0.750)	0.627 (0.601-0.653)	0.465 (cut-off)
30.73	0.706 (0.680-0.730)	0.638 (0.612-0.664)	0.467
30.91	0.650 (0.624-0.680)	0.676 (0.650-0.702)	0.477
32.93	0.572 (0.546-0.600)	0.768 (0.742-0.794)	0.487

*AUC=0.737 (95% CI: 0.686 – 0.788), P =0.000.

Receiver operating characteristic, ROC; DOC, Distance on curve equaling square root of (1-Sen)^2^+ (1-Spe)^2^; AUC, Area under the ROC curve.

**Table 5:** ROC curve for various cut-off levels of LAD/LAV.

**LAD/LVD**	**Sensitivity (95%CI)**	**Specificity (95%CI)**	**DOC**
0.738	0.800 (0.776-0.824)	0.611 (0.587-0.635)	0.438
0.741	0.789 (0.765-0.813)	0.622 (0.598-0.646)	0.433
0.757	0.772 (0.748-0.796)	0.697 (0.673-0.721)	0.379 (cut-off)
0.760	0.750 (0.726-0.774)	0.703 (0.679-0.727)	0.388
0.774	0.700 (0.676-0.724)	0.751 (0.727-0.775)	0.390

*AUC: 0.784 (95% CI: 0.737 – 0.830), *P* =0.000.

Receiver operating characteristic, ROC; DOC, Distance on curve equaling square root of (1-Sen)^2^+ (1-Spe)^2^; AUC, Area under curve.

### Predictive value of LAV or LAD/LVD for LACI in hypertension patients

We divided the study population into two groups according to the cut-off value of LAV (Table 4) or LAD/LVD (Table 5) and analyzed the risk factors of LACI in these two groups. There was an increased incidence of LACI in the LAV over 30.14 group (65.3% vs 30.1%, *P* < 0.001) (Table 6) and in the LAD/LVD over 0.757 group (74.3% vs 24.1%, *P* < 0.001) (Table 7). According to the results of the chi-square test, there was no significant difference between the two LAV groups in sex, diabetes, coronary heart disease or hypertension classification, and also no significant difference between the two LAD/LVD groups in age, diabetes, coronary heart disease or hypertension classification. Hypertension duration was significantly different between the two LAV groups and between the two LAD/LVD groups. Hypertension duration over 20 years accounted for 44.2% of hypertension patients with LAV> 30.14; and 42.6% of hypertension patients with LAD/LVD > 0.757.

**Table 6:** Risk factors predicted by the cut-off value of LAV in the study population.

**Variable**	**LAV<30.14 (n = 166)**	**LAV>30.14 (n = 199)**	***U/χ^2^* Value**	***P* Value**
Age, y	72.63 ± 10.91	77.50 ± 9.70	-4.471	0.000
Gender (M,%)	79 (47.6%)	91 (45.7%)	0.126	0.723
HTN Classification, (n,%)			0.979	0.322
HBP Grade I	69 (41.6%)	93 (46.7%)		
HBP Grade II and III	97 (58.4%)	106 (53.3%)		
HTN time, (n,%)			10.166	0.006
<10 years	57 (34.3%)	46 (23.1%)		
10-20 years	61 (36.7%)	65 (32.7%)		
>20 years	48 (28.9%)	88 (44.2%)		
LACI, (n,%)	50 (30.1%)	130 (65.3%)	44.879	0.000
CHD, (n,%)	116 (69.9%)	120 (60.3%)	3.633	0.057
Diabetes, (n,%)	46 (27.7%)	73 (36.7%)	3.316	0.069

Male, M; Lacunar Infarction, LACI; Hypertension, HTN; Coronary heart disease, CHD; LAV, left atrial volume; LAD, left atrial dimension; LVD, left ventricular dimension.

**Table 7:** Risk factors predicted by the cut-off value of LAD/LVD in the study population.

**Variable**	**LAD/LVD<0.757 (n = 170)**	**LAD/LVD>0.757 (n = 195)**	***t/χ^2^* Value**	***P* Value**
Age, y	74.40 ± 10.22	76.05 ± 10.78	-1.496	0.136
Gender (M,%)	92(54.1%)	78 (40.0%)	7.275	0.007
HTN Classification, (n,%)			1.912	0.167
HBP Grade I	82 (48.2%)	80 (41.0%)		
HBP Grade II and III	88 (51.8%)	115 (59.0%)		
HTN time, (n,%)			6.396	0.041
<10 years	57 (33.5%)	46 (23.6%)		
10-20 years	60 (35.3%)	66 (33.8%)		
>20 years	53 (31.2%)	83 (42.6%)		
LACI, (n,%)	41 (24.1%)	139 (71.3%)	80.828	0.000
CHD, (n,%)	117 (68.8%)	119 (61.0%)	2.417	0.120
Diabetes, (n,%)	52 (30.6%)	67 (34.4%)	0.588	0.443

Male, M; Lacunar Infarction, LACI; Hypertension, HTN; Coronary heart disease, CHD; LAV, left atrial volume; LAD, left atrial dimension; LVD, left ventricular dimension.

Possible influencing factors in hypertension patients with LACI, such as age, sex, CAD, diabetes and cardiac ultrasound indexes, were assessed (Table 8). In univariate analysis, age, LVEF and LAD were significantly associated with LACI. Both univariate and multivariate logistic regression analyses showed that LAV and LAD/LVD were significantly associated with the occurrence of LACI in hypertension patients. In multivariate analysis, LAV was associated with LACI with an odds ratio (OR) of 3.649[95% confidence interval (CI): 1.384-9.621, *P* = 0.009], and LAD/LVD was associated with LACI with an odds ratio (OR) of 8.094 [95% confidence interval (CI): 4.338-15.009, *P* = 0. 000] (Table 8).

**Table 8:** Univariate and multivariate logistic regression model for prediction of lacunar infarction.

**Variable**	**Univariate analysis OR (95% CI)**	***P* Value**	**Multivariate analysis OR (95% CI)**	***P* Value**
Age	1.960(1.151-3.339)	0.013	1.772(0.917-3.423)	0.089
Gender	0.993(0.658-1.498)	0.972	0.698(0.406-1.198)	0.192
CHD	1.134(0.738-1.742)	0.567	1.616(0.948-2.754)	0.078
Diabetes	1.068(0.689-1.654)	0.769	0.834(0.487-1.428)	0.508
LVEF	1.623(1.063-2.479)	0.025	1.566(0.924-2.655)	0.096
IVS	1.494(0.950-2.350)	0.082	2.442(0.726-8.215)	0.149
LVPW	1.225(0.773-1.942)	0.387	0.450(0.130-1.559)	0.208
LAD	3.689(2.392-5.688)	0.000	0.534(0.189-1.514)	0.238
LVD	0.866(0.536-1.400)	0.558	1.074(0.530-2.174)	0.844
LAV	4.371(2.810-6.799)	0.000	3.649(1.384-9.621)	0.009
LAD/LVD	7.810(4.887-12.480)	0.000	8.094(4.338-15.099)	0.000

OR, indicates odds ratio, CI, confidence interval. Coronary heart disease, CHD; LVEF, left ventricular ejection fraction; LVD, left ventricular dimension; IVS, interventricular septal thickness at end-diastole; LVPW, left ventricular posterior wall thickness at end-diastole; LAD, left atrial dimension; LAV, left atrial volume; LVD, left ventricular dimension.

## DISCUSSION

LACI is the most common type of ischemic stroke, resulting from the occlusion of small penetrating arteries that provide blood to the brain's deep structures [11]. When no evidence of small vessel disease is found on histologic examination, an embolic cause is assumed, either artery-to-artery embolism or cardioembolism [11]. In one recent series, 25% of patients with clinical radiologically defined lacunas had a potential cardiac cause for their strokes [12]. Therefore, it is of particular importance to prevent cardiovascular and cerebrovascular complications as early as possible. Hypertensive patients appear to be at increased risk for LACI, with advanced age and current cigarette smoking being the most significant factors among all predisposing factors.

We observed 365 hypertensive participants, and found that the incidence of LACI in the over 20 years history of hypertension group was obviously increased compared with that in the 10-20 years history and below 10 years history group. We also observed that cavity infarction patients with grade I hypertension accounted for 48.1% of the sample, and patients with grade II and above hypertension accounted for 50.2%. Our study indicated that an increased LACI risk was associated with a longer duration of hypertension rather than the hypertension classification, which agreed with previous studies. Hypertension is the most important risk factor for lacunar cerebral infarction. Previous studies also presented a significant association between blood pressure and LACI [13]. Long-term hypertension can cause fibrinoid necrosis, arteriosclerosis, microaneurysm, and hyaline degeneration of arterioles, which result in occlusion, so the incidence of LACI increases [14, 15].

In addition to hypertension, the occurrence of LACI is related to the following factors: age, sex, diabetes and heart diseases. Among these factors, age is related to the duration of hypertension and is significantly increased in LACI patients compared to non-LACI patients. These results indicated that the occurrence of LACI in hypertension patients increased with age. Age is one of the risk factors for hypertension and LACI. Other factors, such as sex, coronary heart disease, and diabetes mellitus, were not significantly different among the different hypertension groups and LACI groups in our study.

In recent years, the size and function of the left atrium have been clinically valued, and many diseases such as stroke [16], atrial fibrillation [17], hypertension [18, 19], acute myocardial infarction [20] and mitral regurgitation [21] can affect the basic function of LA. The relationship between atrial fibrillation and cerebral infarction is clear. There are controversial findings on the relationship between left atrial diameter and ischemic infarction. Broughton ST et al. reported that the association between atrial fibrillation and ischemic stroke was not modified by the presence of left atrial enlargement [22]. Yaghi S et al reported that moderate to severe left atrial enlargement was an independent marker of recurrent cardioembolic or cryptogenic stroke in a multiethnic cohort of ischemic stroke patients [23]. Consistent with previous studies, LAD, LAV,LAD/LVD, IVS, and LVPW in patients with an over 20 years history of hypertension were significantly larger than those in patients with 10-20 years and fewer than 10 years of history in our study, though LVD and LVEF were not different among these groups. However, all cardiac ultrasound indexes were not different between the grade I hypertension group (n= 162) and the grade II hypertension and above group (n= 203). These results suggested that LA enlargement and LV remodeling were positively related to the duration of hypertension rather than the grade of hypertension.

Left atrial enlargement is closely associated with hypertension, and is a risk factor for the occurrence of LACI. However, few studies have focused on the association between left atrial structure and LACI in hypertensive patients. The relationship between left atrial enlargement and the occurrence of LACI in hypertension patients has attracted our attention. Therefore, we observed cardiac ultrasound indexes in hypertensive patients with LACI and found increased LAD, LAV, and LAD/LVD in LACI patients compared to non-LACI patients. Although LAV and LAD/LVD were associated with LACI in hypertensive patients, we still need to know whether these parameters have clinical utility and should be added to risk prediction models. We performed ROC curve analysis to evaluate the predictive value of LAV and LAD/LVD for LACI in hypertension patients and found that the optimal cut-off value for LAV was 30.14 IU/L, and the sensitivity and specificity were 72% and 63%, respectively, while that for LAD/LVD was 0.757 and the sensitivity and specificity were 77% and 70%, respectively. Then we verified the risk factors for LAV and LAD/LVD using the cutoff standard and demonstrated the incidence of LACI in hypertensive patients. The incidence of LACI was significantly increased in the group with LAV or LAD/LVD over the cutoff point. In univariate analysis, LAV, LAD/LVD, LVEF and LAD were predictive for LACI. However, in multiple analyses, only LAV and LAD/LVD were statistically significant. Age was also associated with the prevalence of LACI in univariate analysis but not in multiple analyses. These data suggest that hypertensive patients with advanced age and cardiac hypofunction are more inclined to have LACI. Other factors, such as sex, diabetes, coronary heart disease, myocardial thickness and left ventricular size, showed no predictive value for LACI. Therefore, LAV or LAD/LVD is an independent risk predictor for LACI in hypertensive patients.

This study is a retrospective study with a small sample sizes that was conducted during a limited period, and there might be selection bias in the collection of case data as well. Thus, the results of this study need to be validated by multicenter prospective clinical studies. This study also did not detect serum markers that can reflect the risk of stroke, such as D-dimer, C reactive protein, and brain natriuretic peptide. Therefore, the data were far from being complete. We were unable to provide enough samples to assess all risk factors for LACI.

Cerebral infarction imposes a heavy burden on patients, families, and society. Individuals who experience a LACI are often completely unaware they have suffered a stroke, which often causes lesions in the surrounding brain tissue and confers a greater risk for future major stroke. Therefore, it is important to seek simple and noninvasive cardiac ultrasound indexes to provide new predictors for LACI.

## CONCLUSIONS

In summary, LAD, LAV and LAD/LVD in hypertensive patients, which are significantly related to the incidence of LACI, were independent risk factors for LACI indexes. Ultrasonic evaluation of left atrium size can be helpful for identifying high-risk-group patients who are vulnerable to LACI, and may provide a clinical basis for the primary prevention of LACI in hypertension patients.

## MATERIALS AND METHODS

### Study population

In this study, cross-sectional analysis of 365 patients with hypertension admitted and treated at Shanghai Ninth People’s Hospital between January 2016 and January 2017 was performed. Primary hypertension and grade of hypertension were defined according to the World Health Organization essential diagnostic criteria [24]. LACI was defined as a focal onset of neurological symptoms, with no other explanation, a National Institutes of Health Stroke Scale (NIHSS) score < 8 and no expectation of resulting in dependency (modified Rankin Scale (mRS) score < 3). Stroke was confirmed by an expert panel based on clinical findings and neuroimaging techniques such as MRI and computerized axial tomography (CT scan) [25].

All patients fulfilled the following inclusion criteria but did not meet the exclusion criteria. Patients were included in the study if they met the inclusion criteria: 1) older than 18 years and 2) primary hypertension. The following conditions were exclusion criteria: 1) massive cerebral infarction (isolated ischemic lesions > 20 mm); 2) atrial fibrillation; 3) rheumatic heart valve disease; 4) heart failure and other possible causes of heart thrombosis; and 5) concomitant severe diseases such as liver, kidney, hematopoietic system and endocrine system disorders.

All patients completed a questionnaire and underwent a complete routine clinical and cardiac laboratory evaluation including age, sex, family history physical examination, electrocardiogram, and blood pressure monitoring. Patients were routinely referred to magnetic resonance imaging (MRI) with diffusion-weighted images (DWI) by the neurologist in charge of them after admission to the hospital (Philips Achieva 1.5T MRI, Royal Philips Electronics). Because some patients were unable to undergo MRI due to contraindications such as a pacemaker, 61 patients underwent only CT scanning. Isolated ischemic lesions on MRI, DWI or CT were defined as lacunar infarcts if <20 mm and located subcortically or in the brainstem. Neuroimaging was reviewed by a stroke neurologist (HN) with extensive experience (>15 years) in the interpretation of stroke MRI and CT images. The study protocol was approved by the Shanghai Ninth People’s Hospital Ethics Committee and Research Board. Written informed consent was obtained from all study participants.

### Echocardiography

Transthoracic echocardiography (TTE) was carried out by using a commercially available ultrasound device (ie33, Philips Medical System, Bothell, Washington, USA). Images were obtained from parasternal and apical windows using 2D, M-Mode, and Doppler echocardiography. TTE was performed in all patients, and left ventricular ejection fraction (LVEF) was calculated as (1-end-systolic volume/end-diastolic volume) × 100%. Chamber size was measured by the biplane area-length method on two-dimensional echocardiography. The left atrial diameter was measured at the end of ventricular systole using the M-Mode two dimensional or anteroposterior linear dimension standard technique [26]. The ellipsoid model or Simpson’s rule was used for LA volume estimates.

### Statistical analysis

Statistical analysis was performed using SPSS 17.0 software. Summary statistics were expressed as the mean ± standard deviation for continuous variables, and frequencies and percentages for discrete variables. Fisher’s exact test and the Pearson Chi-square test were used to evaluate categorical variables. A multivariable analysis using multiple logistical regressions was performed for all significant variables in the univariable analysis (hypertension, coronary artery disease, lacunar infarct, baseline NIHSS, and akinetic area on TTE) and for other potentially confounding variables (medication, age, diabetes, baseline blood pressure and systolic ejection fraction). Odds ratios (ORs) 95% confidence intervals (CIs) were calculated for the primary and secondary outcomes. P values <0.05 were considered statistically significant.
